# Supplementary material for: Protocol for Discovering Convergent Molecular Networks across Multi-Omics Datasets Using Network Propagation
Source: ArXiv. 2026 Oct 1:arXiv:2610.01142v1. Preprint. [Version 1] (PMC13648816)
Supplement: Supplementary file 1 [file NIHPP2610.01142v1-supplement-1.pdf]

Table captions

Table S1: Network propagation outputs for various seed set, related to Figure 1B-C.

Table S2. Network edge list and nodes. (A) Edge list for the full network, (B) edge list for the clustered network and (C) node table for the clustered network, related to Figure 2.

Table S3: Enrichment output for the gene set overrepresentation enrichment analysis of the node table above as the input, related to Figure 3.
